# Angiotensin Receptors: Structure, Function, Signaling and Clinical Applications

**DOI:** 10.4172/jcs.1000111

**Published:** 2016-04-08

**Authors:** Khuraijam Dhanachandra Singh, Sadashiva S Karnik

**Affiliations:** Department of Molecular Cardiology, Lerner Research Institute, Cleveland Clinic, 9500 Euclid Avenue, Cleveland, USA

**Keywords:** Angiotensin, AT1 receptor, AT2 receptor, MAS and AngIV binding site, ARBs, RAS

## Abstract

Angiotensinogen – a serpin family protein predominantly produced by the liver is systematically processed by proteases of the Renin Angiotensin system (RAS) generating hormone peptides. Specific cell surface receptors for at least three distinct angiotensin peptides produce distinct cellular signals that regulate system-wide physiological response to RAS. Two well characterized receptors are angiotensin type 1 receptor (AT1 receptor) and type 2 receptor (AT2 receptor). They respond to the octapeptide hormone angiotensin II. The oncogene product MAS is a putative receptor for Ang (1–7). While these are G-protein coupled receptors (GPCRs), the *in vivo* angiotensin IV binding sites may be type 2 transmembrane proteins. These four receptors together regulate cardiovascular, hemodynamic, neurological, renal, and endothelial functions; as well as cell proliferation, survival, matrix-cell interactions and inflammation. Angiotensin receptors are important therapeutic targets for several diseases. Thus, researchers and pharmaceutical companies are focusing on drugs targeting AT1 receptor than AT2 receptor, MAS and AngIV binding sites. AT1 receptor blockers are the cornerstone of current treatment for hypertension, heart failure, renal failure and many types of vascular diseases including atherosclerosis, aortic aneurism and Marfan syndrome.

## Introduction

Renin Angiotensin System (RAS) produces hormonal peptides which signal through cell surface receptors classified as angiotensin receptors. Recent International Union of Basic and Clinical Pharmacology (IUPHAR) review entitled “Angiotensin Receptors: Interpreters of Pathophysiological angiotensinergic stimuli” covered >7255 research articles published in the last 15 years and highlighted enormous development in angiotensin receptor research [1]. The previous review on this topic by de Gasparo et al. [2] is a classic on most cited articles list of Pharmacological Reviews. The current review, also published in Pharmacological Reviews therefore has a high standard to meet in the coming decade.

**Figure 1:**
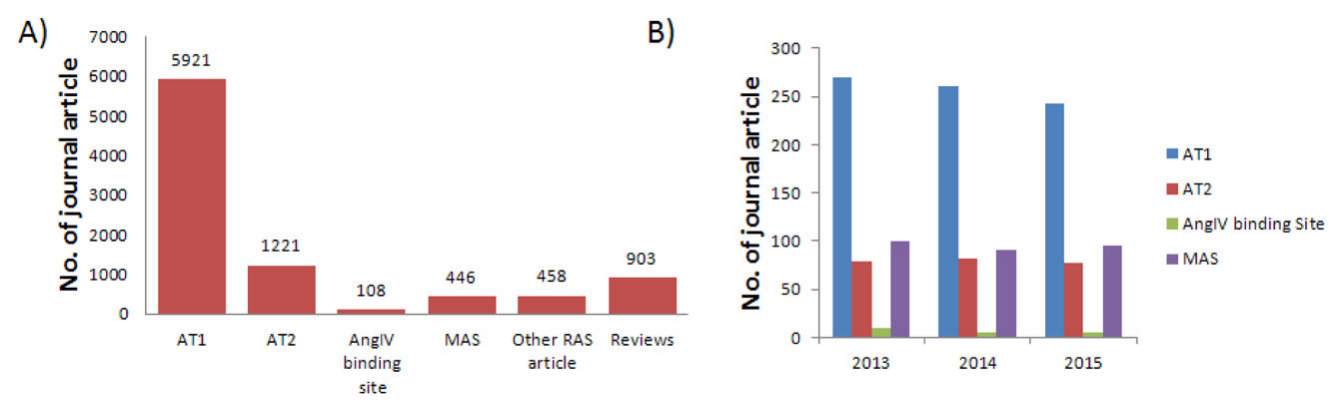
Number of journal article published on angiotensin receptor research. (A) IUBCP XCIX [1] updated up to the end of 2015. (B) Number of journal articles published in years 2013–2015.

Literature covered for the IUPHAR review demonstrated that AT1 receptor studies dominated this research area in the past fifteen years followed by AT2 receptor, MAS and the so called AT4 receptor. Arguably the conflicting results reported on insulin regulated amino peptidase as the cognate receptor for angiotensin IV appears to be a major setback. In contrast, discovery of MAS as a putative Ang (1–7) receptor is a major stimulus of research activity. Continuation of this trend seems to be reflected in our analysis of research literature for years 2013–2015 (Figure 1). AT1 receptor research exceeds the steady number of publication on AT2 receptor, the rising trend for MAS and a clear trending decline for the AngIV binding site (Figure 1).

The review is organized into major sections covering AT1 receptor, AT2 receptor, AT4 binding site, MAS and devoted a section for absence of AT3 receptor in angiotensin receptor nomenclature system. Within each section, advances in structure-function, pharmacology, experimental models, genetics, signalling, expression profile and pathophysiological aspects are discussed with extensive citations provided for >1100 peer reviewed papers. This review is a must read for students and researchers interested in RAS physiology and pathology as well as drug developers.

AT1 receptor– Significant advances took place on almost all aspects of research on AT1 receptor, classically thought to be the sole mediator of all effects of RAS. Recent elucidation of crystal structures of human AT1 receptor bound with the antagonists ZD7155 [3] and Olmesartan [4] facilitates discussion of future mechanistic studies in specific structural details. The crystal structure confirms the postulates 7TM α-helical architecture of AT1 receptor with three extracellular loops (ECL1-3) and three intracellular loops (ICL1-3). The C-terminal region is highly disordered. ECL2 of AT1 receptor exhibits a β-hairpin secondary structure which serves as an epitope for the agonistic autoantibodies in preeclampsia and malignant hypertension [5,6]. AngII bound AT1 receptor crystal structure is currently unavailable.

AT1 receptor blockers (ARBs) are selective non-peptide antagonists in clinical use for the treatment of high blood pressure and are also being examined for various other human cardiovascular disorders [1]. Eight ARBs –azilsartan, eprosartan, candesartan, irbesartan, losartan, telmisartan, olmesartan and valsartan– are available for clinical use. Most of the ARBs excepting telmisartan do not cross the blood brain barrier (BBB) in pre-clinical trials suggesting their efficacy in brain pathological conditions. From the crystal structure and molecular docking simulation critical ligand binding residues (Arg167, Tyr35 and Thr84) identified may facilitate further refinement and development of novel ARBs [3,4]. Inverse agonism of most ARBs is also observed in several studies [7]. Biased agonism of AngII analogs was described and their potential application in heart failure therapy is evaluated in clinical trials.

Significant advances made in defining pathophysiology are extensively reviewed. AT1receptor knockout mice develop polyurea and abrupt vasodepressin signalling observed in the inner medulla [8]. Genetic association studies found that AT1 receptor A1166C (rs5186) polymorphism is associated with essential hypertension, increased aortic stiff [9] and myocardial infarction [10]. Naturally occurring missense variantsmay directly (A163T, T282M and C289W) or indirectly (L48V, L222V and A244S) influence ligand binding and AT1 receptor signals. AT1 receptor signalling is mediated through G-proteins, G-protein independent β-arrestin, reactive oxygen species, non-receptor type tyrosine kinases, small G-proteins, transactivation of receptor tyrosine kinases. Furthermore, interacting scaffold, mechanical stress, heterodimerization; and signalling through phosphorylation, desensitization, and internalization may also be involved. Abnormal activation of AT1 receptor leads to a number of pathophysiologies including cardiovascular remodeling and hypertrophy, vascular inflammation and atherosclerosis, endothelial dysfunction, oxidative stress, extra cellular matrix deposition, insulin resistance, angiogenesis and cancer, autoantibodies and malignant hypertension [1].

### AT2 receptor

The AT2 receptor shares approximately 34% amino acid sequence homology with AT1 receptor [1]. Physiological functions of AT2 receptor are not clearly defined till now, but 15 years of research devoted to this protein have further detailed physiological modulations by AT2 receptor including those promoted by discovery of small molecule agonists and antagonists. Beneficial effects of AT2 receptor have long been unclear due to its low expression in adults. Both AngII and AngIII bind to AT2 receptor with affinity in nano molar range and do not distinguish it from AT1 receptor. Even though the AT2 receptor recognizes the same physiological ligands, the pharmacophore of AT2 receptor is very distinct from that of AT1 receptor. Two non-peptide chemical compounds PD123319 (ditrifluoro acetate) and PD123177 (trifluoro acetate salt) defines the pharmacology and functions of this receptor [11–15]. Recent discovery of a selective AT2 receptor non-peptide agonist, compound 21, may expedite exploration of distinct roles of AT2 receptor in many physiological and pathophysiological states. AT2 receptor became new therapeutic target for the treatment of neuropathic pain. A few molecules like PD123319 [16,17] and EMA401 [18] are in clinical trials but treatment is limited due to poor efficacy and unfavourable side effects.

Expression of AT2 receptors is predominant in distinct brain areas such as the locus coeruleus and [19] and the amygdaloid nucleus [20]. Though, its expression declines after birth, it is expressed at low levels in the normal adult cardiovascular system, adrenal gland, kidney, brain, uterine myometrium and skin [21]. AT2 receptor Knock-out (KO) mouse shows higher blood pressure than wild type animals without any growth abnormalities. Developmental apoptosis of mesenchymal cells is not altered in the AT2 receptor KO mice but increased risk for renal diseases as well as inhibition of pressure natriuresis, vascular hypertrophy and exacerbation of heart failure were observed [22–24]. Beneficial AT2 receptor functions from the knock out mouse study could be protective counteracting blood pressure regulation by the AT1 receptor. Pharmacological modulation of AT2 receptor also suggests its role in antidiuretic and antinatriuretic functions. Studies on genetic polymorphism of this gene revealed their association with mental retardation, ventricular structural changes, metabolic disorders, congenital urinary tract abnormalities etc. [1]. The intra signal transduction process of AT2 receptor is unique among the GPCRs and is different from the AT1 receptor mediated signalling. AT2 receptor signalling involves G-protein, protein phosphatases [Dual specificity protein phosphatase 1(MKp-1), Protein phosphatase 2A (PP2A), Src homology phosphatase-1 (SHP-1)] and scaffolding protein, nitric oxide/cGMP ion channel protein and ion channel protein and constitutive activity (ligand independent activity of AT2 receptor) [1]. The pathological and physiological roles of AT2 receptor include regulation of vascular response, cardiac growth response and fibrosis response in other tissues. The development of agonists and antagonists against AT2 receptor for therapeutic use is crucial and in early stage, hence extensive studies are warranted.

### AT3 receptor

Although existence of AT3 receptor subtype displaying unique pharmacology was reported [25,26] no literature is available confirming the existence of a distinct gene for this receptor in humans to date.

### AT4 receptor

High affinity membrane binding sites for the [125I] AngIV peptide was termed as AT4 receptor in 1995 [27]. They are concentrated predominantly in brain and to different extents in heart, kidney, adrenals and blood vessels. This receptor does not bind the analogues of AngII, [Sar1]AngII, [Sar1,Ile8]AngII, Sar1,Ala8]AngII, Ang(1–7), AngII and the non-peptide inhibitors of AT1 and AT2 receptors losartan, PD123177 and CGD42112A [1]. Histo-autoradiographic mapping studies of AngIV binding site determined the higher concentration of its binding in brain which was then linked to regulation of cognitive sensory and motor functions. Albiston et al (2001) identified [125I] AngIV peptide binding protein as insulin regulated amino peptidase (IRAP, EC 3.4.11.3 also called LNEP for Leucyl-N-exopeptidase) [28] which is a type 2 TM protein of the gluzincin amino peptidase family [29,30].

Several independent observations in recent publications have cast some doubt regarding the identity of IRAP as the only AT4 receptor. For instance, peptide antagonists of AngIV binding sites and small molecule inhibitors of IRAP activity produced divergent physiological effects [31,32]. Moreover, IRAP knockout mice were not altered in their cognitive behavioural response to AngIV. Several other type II membrane proteins have been reported as potential AT4 receptor candidates [33–35]. Therefore, understanding etiology and treatment of memory dysfunctions associated with dementia and degenerative diseases through AT4 receptor is significantly delayed.

### MAS

MAS is a candidate receptor for endogenously produced RAS peptide hormone Ang (1–7) [36]. It remained orphan until the neuropeptide FF was shown to activate G-protein signalling through this receptor. The action of Ang (1–7) through MAS is proposed to be production of arachidonic acid and activation of nitric oxide synthase which may not involve cAMP, IP3 and calcium signalling. MAS exhibits highest expression in brain and testis. Becker et al. (2007) has observed that MAS expression in brain regions is important for cardiovascular regulation [37]. Altered heart rate and decreased blood pressure was observed in KO mice and it was thought to be due to imbalance in the nitric oxide (NO) and reactive oxygen species (ROS) [1]. *In vivo* studies show possible protective role of MAS through Ang (1–7) mediated activation making it an enticing drug target. The pathophysiology of MAS may be related to heart, kidney, vasculature, brain and reproductive organs. Above conclusions are made from *in vivo* physiological and mouse KO studies. Although independent research groups supported some of the findings, extensive pharmacological studies are required to consolidate the conclusion that MAS is Ang (1–7) receptor as well as elucidate its ligand activation mechanism.

## Overall remark

The enormous development in angiotensin receptor research has been addressed on the structure, pharmacological, signalling, physiological and pathophysiological state. Study on AT1 receptor dominates in the field of angiotensin receptor research including the recent solving of its crystal structure which opened new avenues for structure based drug discovery and development. In the near future, we anticipate establishment of structures of other angiotensin receptors. However, research on other angiotensin receptors is in nascent state and extensive study is warranted.
